# The status of Ghanaian community health workers’ supervision and service delivery: descriptive analyses from the 2017 Performance Monitoring and Accountability 2020 survey

**DOI:** 10.12688/gatesopenres.12979.3

**Published:** 2019-08-23

**Authors:** Dan Schwarz, June-Ho Kim, Hannah Ratcliffe, Griffith Bell, John Koku Awoonor-Williams, Belinda Nimako, Easmon Otupiri, Stuart Lipsitz, Lisa Hirschhorn, Asaf Bitton

**Affiliations:** 1 Ariadne Labs, Brigham and Women’s Hospital & Harvard T.H. Chan School of Public Health, Boston, MA, 02215, USA; 2 Division of Global Health Equity, Department of Medicine, Brigham & Women's Hospital, Boston, MA, 02215, USA; 3 Division of General Internal Medicine, Department of Medicine, Brigham & Women's Hospital, Boston, MA, 02215, USA; 4 Policy Planning Monitoring and Evaluation Division, Ghana Health Services, Accra, Ghana; 5 Kwame Nkrumah University of Science and Technology, Kumasi, Ghana; 6 Center for Surgery and Public Health, Brigham and Women's Hospital, Boston, MA, 02215, USA; 7 Feinberg School of Medicine, Northwestern University, Chicago, IL, USA; 8 Center for Primary Care, Harvard Medical School, Boston, MA, USA; 9 Department of Health Care Policy, Harvard Medical School, Boston, MA, USA

**Keywords:** primary health care, community health workers, universal health coverage, Ghana, CHPS

## Abstract

**Introduction: **Community-based services are a critical component of high-quality primary healthcare. Ghana formally launched the National Community Health Worker (CHW) program in 2014, to augment the pre-existing Community-based Health Planning and Services (CHPS). To date, however, there is scant data about the program’s implementation. We describe the current supervision and service delivery status of CHWs throughout the country.

**Methods: **Data were collected regarding CHW supervision and service delivery during the 2017 round of the Performance Monitoring and Accountability 2020 survey. Descriptive analyses were performed by facility type, supervisor type, service delivery type, and regional distribution.

**Results: **Over 80% of CHWs had at least monthly supervision interactions, but there was variability in the frequency of interactions. Frequency of supervision interactions did not vary by facility or supervisor type. The types of services delivered by CHWs varied greatly by facility type and region. Community mobilization, health education, and outreach for loss-to-follow-up were delivered by over three quarters of CHWs, while mental health counseling and postnatal care are provided by fewer than one third of CHWs. The Western region and Greater Accra had especially low rates of CHW service provision. Non-communicable disease treatment, which is not included in the national guidelines, was reportedly provided by some CHWs in nine out of ten regions.

**Conclusions: **Overall, this study demonstrates variability in supervision frequency and CHW activities. A high proportion of CHWs already meet the expected frequency of supervision. Meanwhile, there are substantial differences by region of CHW service provision, which requires further research, particularly on novel CHW services such as non-communicable disease treatment. While there are important limitations to these data, these findings can be instructive for Ghanaian policymakers and implementers to target improvement initiatives for community-based services.

## Introduction

As the world strives to achieve Universal Health Coverage and the Sustainable Development Goals, primary healthcare is foundational to meeting these goals ^1, 2^. Community healthcare systems serve critical roles within strong primary healthcare delivery ^2– 4^. The World Health Organization’s recent guidelines ^5^ for best practices of community health workers (CHWs) offer important guidance to policy makers and program implementers about how to develop strong community health service delivery and support low- and middle-income countries along the path towards universal health coverage. Among other key recommendations, these guidelines highlight the importance of professionally-trained CHWs with clear roles and responsibilities, supported by strong supervision systems to ensure quality service delivery ^5^.

Ghana has a strong history of high-quality community-based primary healthcare delivery, including the development of the Community-based Health Planning and Services (CHPS) in 1994 ^6^, with significant expansion and strengthening of those services over the past 25 years. In recent years, the Ghana Health Service has developed a set of 15 steps and six milestones to guide CHPS implementation across the country ^7, 8^. CHPS service delivery is based on the deployment of Community Health Officers (CHOs) throughout the country in CHPS zones. These CHOs – a type of community health worker in and of themselves ^9^ -- work closely with the Community Health Volunteers (CHVs), who are responsible for home visits, community mobilizations, participation in health outreach services with the CHOs, and household health education ^8^. More detailed descriptions of the roles and responsibilities of CHOs and CHVs are provided in Table 1 and Table 2.

**Table 1.** Roles and responsibilities of Community Health Officers (CHOs) ^8^.

	Community linkage and outreach services	Key tasks
**1**	Health promotion and education	Organize health education and promotion through durbars and home visits; conduct community outreaches, record and report.
2	Disease surveillance	Identify diseases requiring prompt reporting, investigate outbreaks, do surveillance, report according to protocol.
3	Home visits	i. Routine house to house visit: day to day service delivery visits to households and individuals in their homes. ii. Special/Targeted: designate special clients; prepare and conduct home visits. Trace defaulters, follow up patients referred by hospital after discharge, and advise and support clients with non-communicable diseases like diabetes and hypertension. Document and report on these activities.
4	School health	Prepare activities, conduct health education and physical examinations, inspect environment, brief school authorities on findings, and write report.
5	Outreach activities	Prepare and conduct outreach activities; document and report.
6	Managing CHVs	Organize meetings, revise CHAPs, and submit reports.
7	Working with the CHMC	Conduct meetings, write community profiles, draw map of community, and give technical assistance.
	Basic clinical services	Key tasks
**A1. Child health**		
8	Immunization	Education, administration and management of vaccines, recording and reporting.
9	Breastfeeding (BF), growth monitoring, and nutrition	Education, BF support, weighing babies and children, recording, identifying malnourished children, education on prevention of malnutrition.
10	Acute care of infants and children (Integrated Management of Neonatal and Childhood Illness)	History taking; initial assessment; physical examination; identification, classification, and management (jaundice, diarrhea, ARI, fever, measles, ear infection); recording; referral if needed.
**A2. Reproductive health**		
11	Family planning	Counselling on all methods, education on preferred method, administration of method (i.e. condoms, combined oral contractive, injectable, implants), and referral for other or permanent methods.
12	HIV/AIDS and sexually transmitted infections (STIs)	Education, condom use, physical examination, preparing client and using rapid diagnostic test, giving feedback, appropriate management, and referring where necessary.
13	ANC	History taking, identification and management of anemia, malaria in pregnancy, syphilis in pregnancy, implementation of PMTCT activities, counselling pregnant women based on findings, and teaching danger signs in pregnancy
14	Safe emergency delivery and newborn resuscitation	Immediately assess mother, prepare for delivery, monitor labor, deliver baby, resuscitate if baby is not breathing well, and conduct active management of the third stage of labor.
15	Postnatal care (PNC) and essential newborn care	Conduct immediate PNC to mother and baby, educate family on PNC, assess baby and mother at 6 weeks.
**A3. Other clinical services**		
16	Infection prevention	Manage supplies; decontaminate, clean, sterilize, and store instruments appropriately. Dispose of waste properly.
17	Communicable diseases (HIV, malaria, TB)	Recognize signs and symptoms, refer, follow up, conduct home visits for TB. Perform HIV rapid test. Perform malaria rapid test and treat.
18	Non-communicable and chronic diseases (hypertension, diabetes)	Recognize signs and symptoms, refer, follow up, conduct home visits.
19	Neglected tropical diseases	Recognize signs and symptoms, refer, follow up, conduct home visits.
20	Adolescent health	Adolescent-friendly health services, counselling (e.g. FP, STIs and HIVs, nutrition), provision of services, referral as needed, follow-up and home visits.
21	Mental health	Assess and diagnose clients, give appropriate care, and treat if possible.
22	Minor ailments	Assess, diagnose, give appropriate treatment.
23	First aid and home emergencies	Identify signs and symptoms; diagnose and manage shock, snake bite, poisoning, convulsion and seizures, burns, sprains and strains, fractures and dislocations, and epistaxis; and wound dressing.
24	Caring for the Aged	Home visit to the aged to provide education on care and nutrition.
	Resource management	Key tasks
25	Planning	Plan activities monthly and implement them.
26	Logistics management	Request supplies, manage them, manage vaccines well, and keep CHPS compound clean.
27	Financial management	Keep value books, receive completed books, procure utilized books, and receive cash revenues and bank them daily. Collect cheques and bank them; manage petty cash.
28	National Health Insurance Agency	Record and submit NHIS claims.
29	Data collection, reporting, analysis, and use	Collect and record all data; analyses, interpret, and use for decision-making. Ensure that data is entered separately into the DHIMS2 for that particular CHPS zone.

CHV, community health volunteer; CHMC, community health management committee; CHAP, community health action plan; BF, breast feeding; ARI, acute respiratory infection; STI, sexually transmitted infection; ANC, antenatal care; PNC, postnatal care; PMTCT, prevention of mother-to-child transmission; TB, tuberculosis; NTD, neglected tropical disease; FP, family planning; NHIS, National Health Insurance Scheme; DHIMS, District Health Information Management System

**Table 2.** Roles and responsibilities of Community Health Volunteers (CHVs) ^8^.

1	Disease prevention and environmental sanitation	Report any suspected epidemic-prone disease immediately to the community health officer (CHO); educate community members on proper environmental sanitation practices in their communities.
2	Home visiting	Prepare, conduct, and end visits appropriately.
3	Home management of minor ailments (integrated community case management)	Identify and manage fevers, diarrhea at home.
4	Community outreach	Participate, give health education, promote breast feeding, family planning, and wearing and removal of condoms. Equip oneself with home visiting bag.

In 2014, in conjunction with the global One Million Community Health Workers Campaign, the government of Ghana formally launched the National 1 Million CHW Program, with the goal of expanding high-quality community health services throughout the country ^10^. This program was designed to support the pre-existining community health programs that had been built to date ^10^.

In order to address these challenges, a new cadre of health worker, was introduced in the National 1 Million CHW Program ^10^. These CHWs are fully-employed workers, with a salary of approximately $142 USD per month, under the auspices of the Youth Employment Agency ^10^. According to the program design, these CHWs report directly to the CHOs, supporting them to provide first-level health care throughout the communities. Detailed descriptions of the CHW roles and responsibilities are included in Table 3. The program set a goal of deploying over 31,000 CHWs throughout the country between 2014 and 2023 ^10^. By the end of 2019, the goal is to have achieved full rural coverage of the CHW program, involving approximately 28,000 CHWs. As of July 2019, approximately 26,000 have been trained and deployed, the distribution of which can be viewed on the program’s online data dashboard ^11^ and coverage map ^12^.

**Table 3.** Roles and responsibilities of Community Health Workers (CHWs) ^10^.

Condition	Monitor	Counseling and Prevention	Refer and/or Treat	Materials Needed
Case detection, mobilization and referral				
HIV/AIDS	• Assess for danger signs • Monitor for ART adherence • Encourage compliance to ‘Know Your Status’ campaign	• Provide information and awareness about HIV and encourage testing at the health facilities	• Refer HIV+ individuals for ART consultation, if not already participating	
TB	• Assess for danger signs	• Contact tracing • Community / family member sensitization	• Referral of suspected cases of TB • Contact tracing for confirmed cases	
**Manage minor/common ailments and refer more serious afflictions; primary care for simple cases of diarrhea, malaria, acute respiratory diseases, wounds and skin** **diseases; conduct disease surveillance; submit written reports to the SDHT**				
Diarrhea	• Assess for diarrhea	• Provide household counseling on proper sanitary practices, water treatment, and environmental hygiene to reduce onset of diarrhea in their children • Advise on household care of child with diarrhea. • Emphasize continued feeding or increased breast- feeding during, and increased feeding after the diarrheal episode	• Administer ORS Zinc to children (6 months and older) who experience diarrhea and show signs of dehydration but have a MUAC measurement >125 and no indication of edema • Provide caretakers with enough zinc supplements to continue home treatment for 10–14 days	• Oral rehydration salts • Zinc • Chlorine to purify water supply
Fever and Malaria	• Assess for fever • Monitor bednet ownership and correct usage • Ensure coverage of newly pregnant women and newborns with LLINs	• Distribute bednets to households that do not possess them • Replace damaged nets (hole greater than 5cm) and cover new sleeping sites	• Referral of pregnant women and children under 5 who show fever to a facility for proper check-up • Provide ACT (Artesunate AmodiaquineTherapy) for RDT+ and referrals for RDT- in fever cases of children 6 and over • Follow-up of all ill children until recovery after 2 days	• Malaria Rapid Diagnostic Tests • ACTs
Pneumonia	• Assessing fast breathing • Assessing chest in drawing	• Provide household counseling on proper sanitary practices (handwashing, etc.)	• Administer first dose of antibiotic & refer URGENTLY to hospital if suspected severe pneumonia or other very severe disease • If probable pneumonia, give oral antibiotic for five days & soothe the throat and relieve the cough with a safe remedy • Follow-up of all ill children until recovery after two days	• Cotrimoxazole • Paracetamol
**Immunize and provide pre- and post- natal care**				
Neonatal Care	• Complete birth registration • Conduct first visit within 48 hours of birth; bi weekly visits to a household with a newborn child• Monitor EBF • Monitor bednet usage	• Counsel on assessment for life-threatening conditions and physical and mental health of infants • Encourage immunizations • Counsel on EBF for first six months, keeping baby warm, care of umbilical cord, hand-washing with soap, newborn temperature management, and recognizing danger signs	• Refer any newborn children with danger signs to facility	
**Provision of family planning services and referrals**				
Maternal Care & Family Planning	• Enumeration of pregnant women • Monitoring of ANC cards and whether a pregnant woman has received clinical care • Conduct bi-weekly postpartum care visits to assess for danger signs	• Assess iron and folic acid compliance • Review birth plans close to delivery • Referral for delivery at health facility • Distribute condoms and pills • Condom promotion	• Referral for ANC services • Refer to facility for long-term birth control methods	• Measuring tape • Folic acid and iron pills • Condoms • Birth control pills
**Provide education on prevention and management of STDs (syndromic diagnosis)**				
Safe sex education	• Assess at risk sexual behavior, multiple sexual partners, alcohol use, long distance truck drivers	• Educate on condom use • Educate on partner notification of status	• Refer for treatment and counsel on partner notification diagnosis and treatment	
Cholera	• Assess household sanitation and hygiene procedures and conditions • Identify potential cases of cholera • Record all cases in the community and identify water sources that may be contaminated	• Provide household counseling on proper sanitary practices, water treatment, and environmental hygiene • Demonstrate preparation of home-based ORS, hand washing and water filtration • Distribute materials such as soap, aquatabs, and bleach • Distribute ORS	• Refer suspected cases of cholera or other serious cases of water borne illnesses to the health facility • Administer ORS	• Oral rehydration salts • Zinc • Chlorine to purify water supply • Soap
**Community and compound (house to house) level education on primary health care;** **education for health promotion and disease prevention;** **supervise and monitor sanitation efforts**				
Water and Sanitation	• Assess household sanitation and hygiene procedures and conditions • Observe personal hygiene and behavior	• Provide household counseling on proper sanitary practices, water treatment, and environmental hygiene • Demonstrate preparation of home-based ORS, hand washing & water filtration • Distribute ORS	• Refer to facility serious cases of diarrhea or symptoms of cholera or other serious water borne illnesses	• Oral rehydration salts bags. • Chlorine to purify water • Soaps
**Provide nutrition education and care**				
Nutrition	• Assess for nutrition status • Monitor mid upper arm circumference (MUAC) • Conduct growth measurements • Monitor for proper infant feeding	• Promote immediate and exclusive breastfeeding • Promote locally appropriate complementary feeding, highlighting the nutritional value of traditional and locally available foods • Educate on and monitor the use of iodized salt to prevent goiter • Educate on proper food storage techniques	• Referral a child of six months or older to the facility if MUAC measurement <125mm and/or edema are present.	• Infant scales • MUAC bands
**Supervise and monitor community volunteers and TBAs**				
CHVs	• Home visits, community mobilization, participation in health outreach services, health education	• Good and culturally-appropriate behavior, community diplomacy	• Conflict prevention, management and resolution	
TBAs	• ANC cases, deliveries and delivery outcomes	• Personal and environmental hygiene, clean and safe deliveries, hand washing education, clean materials for cord cutting	• Assessment of pregnancies, not to deliver primips, multiple pregnancies, breech • Early referral for difficult labor	

ART, antiretroviral therapy; TB, tuberculosis; SDHT, sub-district health team; ORS, oral rehydration salt; MUAC, mid-upper arm circumference; LLIN, long-lasting insecticidal nets; ACT, artemisinin-based combination therapy; RDT, rapid diagnostic test; EBF, exclusive breast feeding; ANC, antenatal care; STD, sexually transmitted disease; TBA, traditional birth attendants; CHV, community health volunteer.

CHWs are expected to spend 80% of their time in the community, providing these services via household visits. Per the program guidelines, the CHWs are intended to support the CHPS work, and are not supposed to be specifically attached to any hospitals ^8, 10^. In practice however, after the program’s initiation in 2014, anecdotal evidence suggests that many CHWs have been functionally reporting to, or interacting with, facility managers at hospitals.

To ensure the quality of their work, CHWs are expected to meet with their CHO supervisors at least quarterly and also interface with the CHVs during the course of their work, especially in the context of organizing community health-related gatherings and educational campaigns ^8, 10^.

While the policies for training, supervision, and the responsibilities of CHWs are clearly delineated ^10^ -- including twenty-eight weeks of pre-service training and one week update training twice yearly -- there is a paucity of data describing the current state of CHW service scale-up across the country, including how the CHWs’ work relates to the work of the CHOs and CHVs. Given the extensive efforts that have gone into strengthening community-based health services in Ghana, understanding the present status of CHW services is important for policy makers and program implementers to target improvement initiatives for the future.

Here, we present data describing the supervision and activities provided by CHWs throughout the country. These data were collected from the facility surveys done as part of the 2017 round of the Performance Monitoring and Accountability 2020 (PMA2020) national survey ^13^. Given the anecdotal evidence that some CHWs were directly interacting with hospital-level facilities, the survey asked these questions at all facility types, to best characterize the landscape of CHW work nationally.

## Methods

### Survey

The PMA2020 survey is a nationally representative, rapid-turnaround cross-sectional survey of family planning indicators among women of reproductive age (ages 15–49), and water, sanitation, and hygiene indicators among households, in 10 countries ^13^. Using a two-stage cluster design, households were selected to estimate the national modern contraceptive prevalence rate within 3%. In order to better understand access to family planning and primary health care in these countries, data were also collected on health care facilities where women received care. The methods used to collect data from health facilities in the PMA2020 survey have been described in detail elsewhere ^13^. Briefly, health care facilities in each enumeration area were surveyed by trained enumerators, who used mobile data collection technology to interview the heads of facilities and upload the data into a secure cloud server. Data is uploaded as direct responses to the survey tool, as described elsewhere ^13^. We analyzed the PMA2020 survey data collected in Ghana from September 2017 to November 2017 in the 100 enumeration areas surveyed throughout the country ^14^.

In each enumeration area, a census of the public health facilities that serve the enumeration area was conducted to populate the list of survey facilities. Since the survey focused on the primary level of care, the district hospital that serves as the referral facility for all the surveyed facilities was also studied. Facilities of different sizes and levels, from CHPS facilities to health centers and hospitals, were selected to be included in the overall PMA2020 survey sample with the intent to represent the variety of available health facilities in each enumeration area, which are utilized by the nationally representative sample of women of reproductive age.

We explored several aspects of CHW service delivery in Ghana. The PMA2020 survey collected data on whether facilities supported CHWs with supervision and/or supplies (yes/no), what type of facility was reporting CHW data (CHPS/health center/hospital), who at the facilities supervised the CHW (community health officer/public health nurse/midwife/health assistant/physician assistant), and how frequently the CHW was supervised. Frequency of supervision was categorized as days between supervision interactions. If “monthly” was reported, that was categorized numerically as every 30 days.

We also investigated the different types of activities CHWs were involved in, and how these varied by facility type and region. Supervisors were asked about activities and services offered by CHWs from their facility, in reference to CHW activities as defined in the National 1 Million CHW Program documentation ^10^. While not included in the expected scopes of work for CHWs, we also investigated non-communicable disease treatment as a key priority area for potential future service expansion ^8, 10^. All data analyzed had been collected as part of the PMA2020 survey, using the methods previously described.

### Data analyses

Analyses were conducted using descriptive statistics and figures to report on facility-reported supervision and activities of CHWs within the survey. To assess central tendencies and distributions of CHWs and how frequently they were supervised across different facility types we calculated medians, standard deviations (SD), and interquartile ranges (IQRs) by each facility type. We also calculated counts and percentages to determine who supervised CHWs at each facility type, as well as how frequently they were supervised by each facility and supervisor type. Finally, we examined the types of activities CHWs were performing by examining counts and percentages of each activity by facility type and region and created a heat map based on frequency of each activity. As the purpose of this study was descriptive rather than inferential, no null hypothesis testing was conducted. Any missing data are noted in the data tables. No imputation was done for the purposes of this study. Analyses were performed using Stata 15.1 (StataCorp, College Station, TX).

### Ethical statement

This study was approved by the ethical review boards at the School of Medical Sciences / Komfo Anokye Teaching Hospital Committee on Human Research Publications and Ethics (Kumasi, Ghana; protocol CHRPE/AP/740/1.3), Johns Hopkins University (Baltimore, USA; protocol 7238), and Brigham and Women’s Hospital (Boston, USA; protocol 2016P002284). All study participants provided informed, written consent.

## Results

In 2017, 151 healthcare facilities were surveyed and of those, 86 (57%) facilities reported supporting CHWs. The 86 CHW-supporting facilities were distributed across all 10 regions in Ghana and included a mix of hospitals (33.7%), health centers (39.5%), and CHPS facilities (26.7%) ( Table 4).

**Table 4.** Regional distribution of facilities supporting community health workers (CHWs) included in the PMA2020 survey.

Region	Hospitals, n (%)	Health centers, n (%)	CHPS, n (%)	Total, n (%)
Ashanti	6 (37.5)	6 (37.5)	4 (25.0)	16 (100.0)
Brong Ahafo	2 (22.2)	5 (55.6)	2 (22.2)	9 100.0
Central	4 (40.0)	3 (30.0)	3 (30.0)	10 100.0
Eastern	4 (33.3)	3 (25.0)	5 (41.7)	12 (100.0)
Greater Accra	7 (77.8)	2 (22.2)	0 (0.0)	9 (100.0)
Northern	0 (0.0)	3 (100.0)	0 (0.0)	3 (100.0)
Upper East	1 (16.7)	3 (50.0)	2 (33.3)	6 (100.0)
Upper West	0 (0.0)	3 (75.0)	1 (25.0)	4 (100.0)
Volta	3 (37.5)	4 (50.0)	1 (12.5)	8 (100.0)
Western	2 (22.2)	2 (22.2)	5 (55.6)	9 (100.0)
Total	29 (33.7)	34 (39.5)	23 (26.7)	86 (100.0)

CHPS, Community-based Health Planning and Services.

Nationally, there were more CHWs supervised on a per-facility basis at the hospital and health center levels than the CHPS facilities (median number of CHWs per facility: 20, 10, and 4, respectively) ( Table 2). Most CHWs were supervised by CHOs at health centers and CHPS facilities (74% and 78%, respectively), while hospital-based CHW supervision was managed by both CHOs (38%) and Public Health Nurses (62%) ( Table 5).

**Table 5.** Characteristics of community health worker (CHW) distribution and supervision by facility type.

*Distribution of CHWs at each facility type*				
Facility Type	Hospitals	Health centers	CHPS	Total
Number	26	33	23	82
Median	20	10	4	6.5
IQR	31	11	3	16
Minimum	3	3	1	1
Maximum	123	158	12	158
*Who supervises CHWs at each facility type? n (%)*				
Facility Type	Hospitals	Health centers	CHPS	Total
Community health officer	11 (37.9)	25 (73.5)	18 (78.3)	54 (62.8)
Public health nurse	18 (62.1)	2 (5.9)	0 (0.0)	20 (23.3)
Midwife	0 (0.0)	2 (5.9)	4 (17.4)	6 (7.0)
Health assistant	0 (0.0)	0 (0.0)	1 (4.4)	1 (1.2)
Physician assistant	0 (0.0)	5 (14.7)	0 (0.0)	5 (5.8)

* Missing CHW count data on 4 sites. CHPS, Community-based Health Planning and Services; IQR, interquartile range.

Nationally, there was considerable variability in the frequency of supervision interactions between CHWs and their supervisors, and these data show that the majority (55.8%) of CHWs interacted with their supervisors approximately once per month ( Table 6). An additional 25.6% of CHWs interacted with their supervisors more than once per month, meaning than over 80% of CHWs described in these data had at least monthly supervision interactions ( Table 6). The frequency of interactions did not seem to vary substantially by facility or supervisor type. CHWs based at hospitals, health centers, and CHPS all interacted with their supervisors at approximately the same frequency (median number of days between interactions: 30, 30, and 30, respectively) ( Table 7). The frequency of supervision interactions did not differ between types of supervisors (public health nurses, CHOs, midwives), with a median of 30 days between interactions for all supervisor types, except for the single Health Assistant supervisor included in the sample (7 days) ( Table 7).

**Table 6.** Frequency of community health worker (CHW) supervision interactions.

Days between interactions	Number	Percent
Daily	5	5.8
3	1	1.2
7	14	16.3
14	2	2.3
30	48	55.8
60	4	4.7
90	6	7.0
120	6	7.0
Total	86	100.0

**Table 7.** Frequency of community health worker (CHW) supervision interactions by facility and supervisor types.

*Number of days between supervision of CHWs by facility*					
Facility Type	Number	Median	IQR	Minimum	Maximum
Hospitals	29	30	23	1	120
Health centers	34	30	0	1	120
CHPS	23	30	0	3	120
Total	86	30	16	1	120
*Number of days between supervision of CHWs by supervisor type*					
Supervisor Type	Number	Median	IQR	Minimum	Maximum
Community health officer	54	30	0	1	120
Public health nurse	20	30	23	1	120
Midwife	6	30	0	30	30
Health assistant	1	7	0	7	7
Physician assistant	5	30	0	1	120
Total	86	30	16	1	120

CHPS, Community-based Health Planning and Services; IQR, interquartile range.

There was wide variability in the types of services delivered by CHWs, by both facility type and region, as described in Table 8 and Table 9. Of the activities that are expected to be delivered by CHWs according to the National 1 Million CHW Program policies ^10^, some services, such as community mobilization, health education, and outreach for loss-to-follow-up, were delivered by over three-quarters of all CHWs ( Table 8). In contrast, other services, such as mental health counseling and postnatal care were much less common, being delivered by less than one third of CHWs nationally. Notably, while not included in the expected scope of work by national guidelines, 22.4% of CHWs were reported to be providing non-communicable disease treatment services. Regionally, there was great variation in service delivery, with some services, such as active case finding or immunizations, being delivered by all CHWs in one region but not delivered by any CHWs in other regions ( Table 9).

**Table 8.** Community health worker (CHW) activities by facility type.

CHW activity	Overall *		Hospitals		Health centers & clinics		CHPS	
	No.	%	No.	%	No.	%	No.	%
Community mobilization	75	88.2	24	82.8	33	97.1	18	81.8
Health education	67	78.8	22	75.9	31	91.2	14	63.6
Outreach for loss to follow-up	65	76.5	21	72.4	29	85.3	15	68.2
Disease surveillance	61	71.8	19	65.5	27	79.4	15	68.2
WASH counseling	58	68.2	18	62.1	26	76.5	14	63.6
Enrollment in facility	56	65.9	20	69.0	26	76.5	10	45.5
Active case finding	54	63.5	17	58.6	24	70.6	13	59.1
FP counseling	47	55.3	14	48.3	24	70.6	9	40.9
FP Provision	45	52.9	12	41.4	21	61.8	12	54.5
ANC counseling	42	49.4	13	44.8	21	61.8	8	36.4
C-IMCI-iCCM	35	41.2	7	24.1	20	58.8	8	36.4
Immunization	34	40.0	15	51.7	12	35.3	7	31.8
Directly observed therapy for TB	32	37.6	11	37.9	16	47.1	5	22.7
Mental Health Counseling	25	29.4	9	31.0	12	35.3	4	18.2
Postnatal care	19	22.4	6	20.7	11	32.4	2	9.1
Non-communicable disease treatment ^	19	22.4	6	20.7	10	29.4	3	13.6

* Data missing on one facility. ^Not included in the national CHW guidelines. CHPS, Community-based Health Planning and Services; FP, family planning; TB, tuberculosis; ANC, antenatal care; C-IMCI-iCCM, Community Integrated Management of Childhood Illnesses – Integrated Community Case Management; WASH, water, sanitation and hygiene.

**Table 9.** Community health worker (CHW) activities by region.

	Overall *		Ashanti		Brong Ahafo		Central		Eastern		Greater Accra		Northern		Upper East		Upper West		Volta		Western	
	No.	%	No.	%	No.	%	No.	%	No.	%	No.	%	No.	%	No.	%	No.	%	No.	%	No.	%
Community mobilization	75	88.2	15	100	7	77.8	10	100	11	91.7	8	88.9	3	100	6	100	4	100	7	87.5	4	44.4
Health education	67	78.8	11	73.3	8	88.9	10	100	9	75	5	55.6	2	66.7	6	100	4	100	8	100	4	44.4
Outreach for loss to follow-up	65	76.5	10	66.7	6	66.7	10	100	11	91.7	7	77.8	3	100	6	100	4	100	7	87.5	1	11.1
Disease surveillance	61	71.8	13	86.7	8	88.9	7	70	10	83.3	6	66.7	2	66.7	6	100	4	100	5	62.5	0	0
WASH counseling	58	68.2	9	60	7	77.8	10	100	10	83.3	4	44.4	3	100	4	66.7	3	75	6	75	2	22.2
Enrollment in facility	56	65.9	8	53.3	7	77.8	7	70	8	66.7	6	66.7	3	100	6	100	3	75	8	100	0	0
Active case finding	54	63.5	12	80	8	88.9	4	40	8	66.7	6	66.7	2	66.7	5	83.3	4	100	5	62.5	0	0
FP counseling	47	55.3	8	53.3	3	33.3	8	80	8	66.7	1	11.1	3	100	3	50	3	75	7	87.5	3	33.3
FP Provision	45	52.9	4	26.7	4	44.4	8	80	6	50	1	11.1	3	100	1	16.7	3	75	7	87.5	8	88.9
ANC counseling	42	49.4	7	46.7	4	44.4	6	60	6	50	1	11.1	3	100	5	83.3	3	75	6	75	1	11.1
c-IMCI-iCCM	35	41.2	7	46.7	3	33.3	4	40	8	66.7	1	11.1	2	66.7	3	50	2	50	5	62.5	0	0
Immunization	34	40	6	40	3	33.3	2	20	2	16.7	9	100	0	0	1	16.7	1	25	6	75	4	44.4
Directly observed therapy for TB	32	37.6	7	46.7	4	44.4	2	20	8	66.7	2	22.2	1	33.3	2	33.3	1	25	5	62.5	0	0
Mental Health Counseling	25	29.4	4	26.7	1	11.1	6	60	5	41.7	0	0	1	33.3	1	16.7	2	50	5	62.5	0	0
Postnatal Care	19	22.4	4	26.7	2	22.2	3	30	2	16.7	0	0	0	0	2	33.3	1	25	5	62.5	0	0
Non-communicable diseases ^	19	22.4	3	20	1	11.1	3	30	4	33.3	0	0	1	33.3	2	33.3	1	25	3	37.5	1	11.1

* Data missing on one facility. ^ Not included in the national CHW guidelines. FP: family planning, TB: tuberculosis, ANC: antenatal care, C-IMCI-iCCM: Community Integrated Management of Childhood Illnesses – Integrated Community Case Management, WASH: water, sanitation and hygiene

## Discussion

In Ghana, where there is a long-standing commitment to quality community-based primary healthcare, the 2014 National 1 Million CHW program was designed to strengthen the pre-existing community-based service provision. To date, however, there is scant data to understand the success of the program implementation. We have presented data that show variability in both supervision and the CHW activities provided across the country. Additionally, these data show very clearly that, while not designed to be posted to hospitals, hospital-supervised CHWs are common across the country. The details of these data offer several important insights to program implementers and policy makers for the future of strong community-based primary healthcare services in Ghana.

The variability in the frequency of supervision interactions between CHWs and their supervisors is notable, in light of national ^10^ and global ^5, 15^ guidelines that aspire to consistent, frequent supervision systems for CHWs to ensure quality service delivery. The variability seems to be agnostic of facility type or supervisor type, and over 80% of the CHWs described here were reported to be interacting with their supervisors at least monthly, which is much more frequently than the quarterly goals set forth in the National CHW Program guidelines ^10^. While more frequent supervision is likely beneficial, this reported variability in frequency of interactions offers a clear area for standardization throughout the program. Additionally, even amongst the CHW-supervisor pairs that are meeting national goals, it would be informative to investigate the ideal frequency of supervision in order to optimize limited resources.

Our data show considerable variability in the type of activities performed by the CHWs, and the degree of availability of each activity, across the different regions of the country. While this survey inquired about only a sample of the expected services included in the national guidelines ^10^, it is clear that many expected activities are not yet being provided by CHWs, or only minimally provided in certain regions. Only three activities – community mobilization, health education, and outreach for loss to follow-up patients – were reported to be provided by the CHWs affiliated with more than three-quarters of surveyed facilities nationally, and even these were not universally available throughout all regions. Multiple other services that are included in the national guidelines, including antenatal care (ANC) counseling, community-based integrated management of childhood illness, immunization services, mental health counselling, and post-natal care, were reported to be provided by less than half of CHWs nationally, and far fewer in some regions.

At the regional level, we also found variability in service provision, with some regions’ facilities reporting much higher provision of CHW activities than others. In particular, the Western region reported especially low rates of CHW services provided, with all activities except family planning provision (88.9%) being provided by CHWs affiliated with less than half the facilities, and six expected activities being provided by no facility at all. The Greater Accra region also had lower provision rates of many activities, which may be related to differential implementation of the CHW program within the larger urban area, where services might be provided by other actors and facility types, unlike the more remote areas.

Our data show evidence of an expanded role for CHWs, beyond that specified in the national guidelines. All regions except the Greater Accra region reported CHW provision of non-communicable disease treatment. While these data only describe what the facility managers reported, and thus cannot provide insights into the details of these non-communicable disease services, nor the technical quality of their provision, this is an important finding. Given that these are not included in the national CHW guidelines, this demonstrates that there is at least some implementation of novel service delivery throughout the country. Some of these activities may be provided in the context of local pilot programs or community-based programs, although our survey data are not specific enough to elucidate those details. Regardless, given that non-communicable diseases are priorities for the national health sector ^8^, this finding warrants further investigation to better understand the feasibility of CHWs providing these services at a high level of quality, and planning for potential inclusion in the national program in a more standardized manner.

Finally, our data show that, in eight of the ten regions, at least some CHWs are supervised by CHOs who operate from hospitals. These CHOs have been assigned CHPS zones in which they work with the CHWs, as mentioned on the data summary page ^12^. Given that the program is intended to support the CHPS work, and that the CHWs are supposed to spend more than 80% of their time in the community, this finding has important implications for the future of the program. Notably, it is plausible that the multiple types of community health cadres, with often-times overlapping or conflicting sets of job descriptions and service delivery guidelines, may have contributed to this phenomenon of CHWs being supervised by CHOs at hospitals. The new guidelines for CHPS were released in 2016 ^8^, which may help to clarify scopes of work among the different cadres supporting community health activities throughout the country.

## Limitations

Our data have several important limitations. First, they are descriptive data only, which were collected in the process of the PMA2020 survey, which is not explicitly designed to study CHW activities. Thus, their level of detail is limited, and further investigation is required to better characterize and understand the aforementioned findings.

Second, these data are from facility manager reports, who may have limitations in their knowledge, which may impact the quality and accuracy of these data. Relatedly, it is not possible for us to determine what percentage of the entire CHW population is accurately reflected in these data; there may be many CHWs who are not in frequent contact with these managers and thus not well-represented by these data. Additionally, since these data are all from facility managers, who may have their own inherent biases, it is quite possible that some of these data represent over-estimates of CHW supervision and activities.

Third, given that the methodology of the PMA2020 sampling strategy is not designed around CHW staffing, the collected data may not be optimal in all regions of Ghana, and importantly do not reflect the new 16-region geographical distribution, which was expanded from the prior 10-region distribution in early 2019. The new 16-region geographical distribution can be seen on the Ghanaian Embassy site.

Finally, our survey inquired very specifically about “community health workers” during each facility survey, but given the multiple cadres involved in community health-related services throughout the country (including, for example, CHOs and CHVs ^9^), it is plausible that some survey respondents may have provided answers that were not exclusively about the CHWs affiliated with their facility. Thus, our data may represent information about other community health-related cadres in Ghana. Further research and program planning should include survey methods to more explicitly differentiate CHWs from the other cadres, to ensure that the correct conclusions are attributed to the appropriate cohort of health workers.

## Conclusions

We have presented descriptive data summarizing the current status of CHW supervision and activities in Ghana. These data provide policy makers and program implementers helpful insights to inform targeted improvement initiatives throughout the country. Furthermore, these data can help to better inform ongoing monitoring and evaluation strategies of community health programming in Ghana. Other countries that utilize the PMA2020 survey methodology, or comparable survey methods, may consider using similar survey techniques, as described here, to better understand their national community health programming.

## Data availability

### Underlying data

All data used in this study are available via the PMA2020 website. Per the data use guidelines of the PMA2020 databases, all PMA2020 datasets are free to download and use, although users are required to register for a PMA2020 dataset account. This is to ensure that data use can be appropriately tracked by the PMA2020 database managers. The request form must include a brief description of the research or analysis that the user would like to conduct using the requested data. If the research question is not clear, the database managers of PMA2020 may follow-up for further clarification. Once users are granted access, a zipped folder with the compressed dataset, brief user notes, and survey questionnaires will be made available to the user. All data sets will be de-identified. Users can download the codebooks as well.
