# Supplementary material for: The status of Ghanaian community health workers’ supervision and service delivery: descriptive analyses from the 2017 Performance Monitoring and Accountability 2020 survey
Source: Gates Open Res. 2019 Aug 23;3:1468. Originally published 2019 May 20. [Version 3] doi: 10.12688/gatesopenres.12979.3 (PMC6600081; doi:10.12688/gatesopenres.12979.3)
Supplement: Peer review history

# Peer review: Reviewer response for version 2

**DOI:** 10.21956/gatesopenres.14143.r27340

**Published:** 2019-06-27

**Authors:** Henry B. Perry

**Affiliations:** 1 Department of International Health, Johns Hopkins Bloomberg School of Public Health (JHSPH), Baltimore, MD, USA

This is a valuable and important study. We need more independent national assessments of CHW programs published in the peer-reviewed literature, so this is a welcome addition.

- I would like to see a better description of the new CHW cadre in Ghana. How many are there now? How much training did they receive? Are they paid?
- In the limitations section, there should also be an emphasis on the fact that there is no way to know what percentage of the entire CHW cadre is being supervised since the survey only picks up reports from a representative sample of facility managers. There may be a significant percentage of CHWs that are not in contact with a facility or a supervisor and therefore may not be supervised. We can’t tell from the data at hand.
- There should be an acknowledgement that CHOs are also considered to be CHWs themselves since they work both at CHPS health posts and in the community outside of the CHPS health post. It might be good to reference the national CHW case study of Ghana that is reported in Perry *et al. *(2017 ^1^).

I confirm that I have read this submission and believe that I have an appropriate level of expertise to confirm that it is of an acceptable scientific standard.

## References

[1] : Case Studies of Large-Scale Community Health Worker Programs: Examples from Afghanistan, Bangladesh, Brazil, Ethiopia, Niger, India, Indonesia, Iran, Nepal, Pakistan, Rwanda, Zambia, and Zimbabwe. *United States Agency for International Development*.2017; Reference source

# Response

**Published:** 2019-08-13

**Authors:** Dan Schwarz

**Affiliations:** Brigham and Women's Hospital, USA

We are grateful to Dr. Perry for his thoughtful comments. In version three of this manuscript, we have attempted to address these comments, adding clarifying text throughout the manuscript, additional citations, and correcting prior typographical errors.

# Peer review: Reviewer response for version 2

**DOI:** 10.21956/gatesopenres.14143.r27343

**Published:** 2019-06-25

**Authors:** Madeleine Ballard

**Affiliations:** 1 Community Health Impact Coalition, Berlin, Germany; 2 Icahn School of Medicine at Mount Sinai, New York City, NY, USA

The authors are to be commended for their succinct yet comprehensive overview of Ghana's multi-layered community health delivery structure and clear presentation of descriptive statistics regarding supervision and service delivery.

 A few small edits worth considering:

1. The 2016 CHPS Policy notes that there had been confusion about the basic minimum service package due to conflicting messages from different levels of the health system. While the 2016 policy clearly defines a minimum package of services, it may be worth noting in text that this earlier confusion may be one reason for the variability noted and that the newly issued guidance may *potentially* have rectified some of this variability in the years between the survey and now.
2. In many countries, CHWs are only attached to the lowest level of care (e.g. community clinic), not district hospitals. It would be worth noting for the reader the policy in Ghana - are CHWs supposed to be attached to district hospitals or is that a quirk of implementation?
3. In the limitations section, it is noted that the quality and accuracy of the data may have suffered due to incomplete knowledge on the part of facility managers who provided it. Given the interests and responsibilities of the managers, would it not also be fair to consider the strong possibility of bias and potential that - if anything - the supervision frequency was overestimated rather than underestimated?
4. There are a few small typos:
  - Table 1: Incorrect bolding of #1.
  - Table 1: Inconsistent capitalization in key tasks of item 3.
  - Table 3: Inconsistent use of periods/full stops throughout the table.
  - Table 3: Inconsistent capitalization in "5 days & soothe the throat".
  - p. 12: Missing period/full stop "attributed to the appropriate cohort of health workers."

I confirm that I have read this submission and believe that I have an appropriate level of expertise to confirm that it is of an acceptable scientific standard.

# Response

**Published:** 2019-08-13

**Authors:** Dan Schwarz

**Affiliations:** Brigham and Women's Hospital, USA

We are grateful to Dr. Ballard for her thoughtful comments. In version three of this manuscript, we have attempted to address these comments, adding clarifying text throughout the manuscript, and correcting prior typographical errors.
